# Global Health *in* Action: a call for pictures

**DOI:** 10.3402/gha.v9.30772

**Published:** 2016-01-04

**Authors:** Julia Schröders, Caroline Sutton

**Affiliations:** 1 Managing Editor Global Health Action, Epidemiology and Global Health Unit, Department of Public Health and Clinical Medicine, Umeå University, Umeå, Sweden; 2 Editorial Director and Co-Founder, Co-Action Publishing, Järfälla, Sweden

*Global Health Action* (*GHA*) has published more than 550 articles since its launch in May 2008. *GHA* was truly the first Open Access global health journal. From 2008 till date, the journal has remained true to its aim to ‘contribute to fuelling a more concrete, hands-on approach to global health challenges’ (1). Published content fulfills the founders’ expectation of addressing a global health agenda in a creative, constructive, and relevant manner with a strong focus on policy and implementation (2). Every day, we publish articles that document – through text and data – the various aspects of global public health challenges that unfortunately still feed upon the widening gap between the winners and losers of globalization.

The research published in *GHA* is compelling. Yet, we believe we can further augment our usual calls for *written* papers to generate new knowledge and evidence with powerful images that further drive home the need to bridge gaps between existing knowledge and the implementation of relevant findings. As the old saying goes, *a picture is worth a thousand words*. In light of this, we now invite our readers to submit striking photographs that literally picture ‘Global Health *in* Action!’ The focus of this very first call for pictures is *Action!* We encourage photographs of actions, movements, or other kinds of engagements that help to better the lives of the most disadvantaged and vulnerable around the globe. Consistent with our vision and mission, we are *looking for* concrete, hands-on approaches to global health challenges, that is, pictures and images that address and deliver a powerful reality about Global Health *in* Action, evoking the quote from Sir Ahmed Salman Rushdie that ‘A photograph is a moral decision taken in one eighth of a second’ (3).

Submission of photographs is free of charge and open to anyone from any country. Authors who have previously published with us are especially encouraged to submit photographs related to their earlier publication in *GHA*. Each submission should be accompanied by a write-up of up to 300 words that detail the context for the image (i.e. who or what is featured in the photograph, when and where was it taken, its significance to the photographer, the photograph's relation to the call Global Health *in* Action, or any other information the photographer deems important). The photograph submitted should not have been used or published previously, neither in print nor online. In the event of an identifiable individual being photographed, the photographer should obtain consent from him or her, or their next of kin. Photographs – color or monochrome – should be in a common image file format (e.g. *jpeg*, *gif*, *png*) and if possible not exceed 5 MB. Digital manipulation of the photograph may be permitted but overly processed photos will be disqualified.

A select group from the *GHA* editorial team and Co-Action Publishing will judge all submissions. The winner will be recognized with a certificate and profile in the journal and through various other channels. Please submit your image and word document to Pictures@globalhealthaction.net and use the subject line: ‘Call for pictures – Global Health *in* Action’. Detailed guidelines and instructions – including ethical considerations – for submitting photographs can be found on the journal website in the Author Guidelines section. Photographs will be accepted until 31 October 2016. Submissions and publication are free of charge.

Thank you for sharing your pictures with us!

*Julia Schröders* Managing Editor Global Health Action Epidemiology and Global Health Unit Department of Public Health and Clinical Medicine Umeå University Umeå, Sweden *Caroline Sutton* Editorial Director and Co-Founder Co-Action Publishing Järfälla, Sweden
